# Applications of Metal-Organic Frameworks in Food Sample Preparation

**DOI:** 10.3390/molecules23112896

**Published:** 2018-11-06

**Authors:** Natalia Manousi, George A. Zachariadis, Eleni A. Deliyanni, Victoria F. Samanidou

**Affiliations:** 1 Laboratory of Analytical Chemistry, Department of Chemistry, Aristotle University of Thessaloniki, Thessaloniki 54124, Greece; nmanousi@chem.auth.gr (N.M.); zacharia@chem.auth.gr (G.A.Z.); 2 Division of Chemical Technology, Department of Chemistry, Aristotle University of Thessaloniki, Thessaloniki 54124, Greece; lenadj@chem.auth.gr

**Keywords:** metal-organic frameworks, MOF, sample preparation, HPLC, GC, food samples

## Abstract

Food samples such as milk, beverages, meat and chicken products, fish, etc. are complex and demanding matrices. Various novel materials such as molecular imprinted polymers (MIPs), carbon-based nanomaterials carbon nanotubes, graphene oxide and metal-organic frameworks (MOFs) have been recently introduced in sample preparation to improve clean up as well as to achieve better recoveries, all complying with green analytical chemistry demands. Metal-organic frameworks are hybrid organic inorganic materials, which have been used for gas storage, separation, catalysis and drug delivery. The last few years MOFs have been used for sample preparation of pharmaceutical, environmental samples and food matrices. Due to their high surface area MOFs can be used as adsorbents for the development of sample preparation techniques of food matrices prior to their analysis with chromatographic and spectrometric techniques with great performance characteristics.

## 1. Introduction

Sample preparation is the most challenging step of the analytical procedure for the analysis of most samples. An appropriate sample preparation technique should not only be simple, fast and economical, but it should also be in regard with the main principles of green chemistry [1,2]. Solid-phase extraction (SPE) is a well-established sample preparation technique; which however shows some fundamental disadvantages, such as including complicated and time-consuming steps, as well as requiring large amounts of sample and organic solvents. As a result, many novel techniques have been developed [1,2,3,4,5]. Nowadays, a trend in analytical chemistry is to develop new sorbents either for the well-established SPE procedure or for the novel microextraction procedures, which have been gaining more and more attention [1]. New sorbents such as molecular imprinted polymers (MIPs), carbon-based nanomaterials, carbon tubes, graphene based materials, or metal-organic frameworks (MOFs) are becoming more and more popular [6,7,8]. Metal-organic frameworks are a new class of hybrid organic inorganic supramolecular materials, which are based on the coordination of metal ions or clusters with bi- or multidentate organic linkers [9,10]. Metal-organic frameworks became popular in 1995, when Yangi and Li reported the synthesis of a metal-organic framework containing large rectangular channels [11] What makes the use of MOF materials so promising is the fact that they bare great physical and chemical properties, such as their high surface areas (up to 10,000 m^2^/g), in addition with their tunable pore size and functionality, and can act as hosts for a variety of guest molecules. Some of MOFs great properties are luminosity, flexibility of their structure, charge transfer ability from the ligand to the metal or from the metal to the ligand, thermal stability, properties that include electronic and conducting effects and pH-sensitive stability [11,12].

For the synthesis of MOF materials many alternative ways have been proposed. The most famous method is the solvothermal method, which is normally performed in an autoclave with high temperature and pressure and with the use of an organic solvent at its boing point (typically dialkyl formamides, alcohols and pyridine) [13]. Other synthetic methods that have been applied for MOF materials include microwave, electrochemical, mechanochemical, ultrasonic, high-throughput syntheses and more novel techniques include post-synthetic deprotection [12].

Therefore, MOFs have been applied in many different scientific fields and their most famous application is for storage of gas fuels such as hydrogen and methane [14]. Other applications of MOFs include gas separation proton, electron, and ion conduction, capture of carbon dioxide and organic reaction catalysis applications [15] Biomedical applications of MOFs include biomedical imaging, disease diagnosing, drug delivery, biosensing and magnetic resonance imaging [15,16,17,18,19]. In the field of analytical chemistry many different applications of MOF materials have been reported. In 2006, Chen et al. used for the first time MOF-508 material as stationary phase in a packed column in gas chromatography (GC) [20]. After that, some other MOFs have been used in packed GC columns [21,22,23]. Moreover, MOFs have been used as stationary phases in HPLC columns both for normal-phase and for reversed-phase high performance liquid chromatography (HPLC) applications [24,25,26]. However, the most popular applications of the use of MOFs in analytical chemistry are in the field of sample preparation as absorbents for the extraction of a wide range of analytes in different matrices [27].

In the last few years the very promising properties of MOF materials, such as the high surface area, made MOF ideal materials to be used as absorbents for sample preparation to meet various separation needs for many different compounds including either organic compounds or metal compounds from a wide range of matrices, such as environmental samples, food samples, drinking water etc. Typical examples of MOF materials that have been used as absorbents for sample preparation are MOF-199, MOF-5(Zn), ZIF-8, and MIL-53(Al). Most of the times, the mechanism of absorption may be due to the π–π stacking interaction between the MOF material and the analytes because of the presence of sp^2^ hybridized carbons [15].

Another interesting category of materials are metal organic frameworks derived nanoporous carbons, which are also useful materials for sample preparation. These materials have properties similar to MOFs and therefore they can form π-interactions between them and benzene rings of the target analytes. Direct carbonization or carbonization/polymerization after impregnation of MOF carbon precursors with furfuryl alcohol can lead to the formation of those materials. As a result, MOF derived nanoporous carbons are also considered as useful adsorbents for sample preparation [28].

Herein, we aim to point out the applications of MOFs, which are reported in the literature which include the use of metal-organic compounds and their derived carbons, as absorbents in combination with dispersive sample preparation techniques, magnetic sample preparation techniques, in-tube sample preparation techniques and on-line sample preparation techniques for the analysis of complex food samples, such as milk, tea and beverages, fruits and vegetables, meat, chicken, fish etc.

## 2. Food Matrices

**Figure 1:**
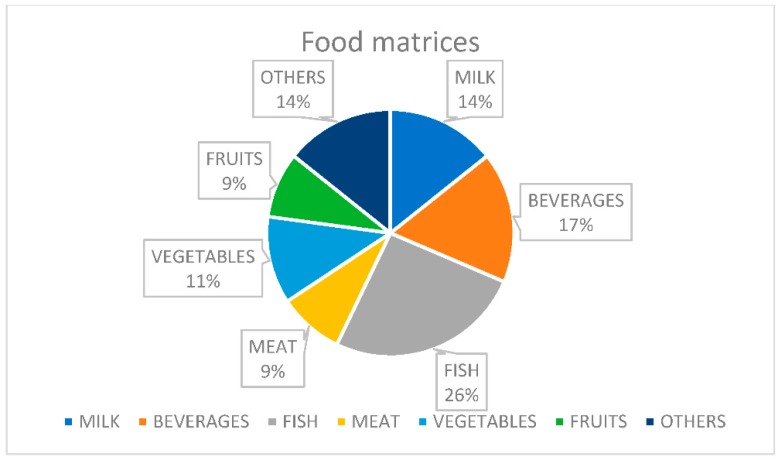
Food matrices treated with MOFs for analytical purposes.

Metal-organic frameworks have been used for many different food matrices (Figure 1).

### 2.1. Milk Samples

Milk is an important and well-studied matrix because its quality depends directly on the nutrition and medication that is given to the animals that produce it. A wide range of antibiotics have been examined in milk samples and many analytical methods have been developed for the determination of those compounds in milk samples based on the legislation and the maximum residue limits. MOF materials have been used as absorbents for the extraction of different kind of analytes such as sulfonamides, penicillins, tetracyclines, etc. The wide sulfonamide class of antibacterial compounds has been widely examined due to their excessive use in veterinary practice [29,30]. In 2017 Jia et al., synthesized a novel hybrid MOF/graphene oxide (GO) material for the dispersive micro-solid phase extraction (d-μSPE) prior to the determination of trace sulfonamides in milk with ultra-high pressure liquid chromatography-tandem mass spectrometry (UHPLC-MS/MS). For this purpose, GO was synthesized and then MIL-101(Cr)@GO material was formed with the hydrothermal method by mixing the graphene oxide with hydrofluoric acid, chromium(III) nitrate nonahydrate and terephthalic acid. For milk sample preparation, ethyl acetate was added to the sample, and the mixture was centrifuged. Accordingly, the supernatant was evaporated, re-dissolved in deionized water and pH was adjusted to 5. Then 5 mg of the material was dispersed in the sample and vortex mixing took place for 20 min. When extraction was finished, the material was separated from the liquid with centrifugation and desorption of the analytes was achieved with 5% ammonia-methanol in ultrasonic bath for 10 min. Detection limits ranged between 0.012 and 0.145 μg/L and recoveries ranged between 79.83% and 103.8%. The developed method was rapid and easy, and the composite MOF material can be implemented for milk analysis and its use can be extended for other non-volatile analytes [31]. Table 1 summarizes the use of different MOFs in various food matrices as well as some analytical characteristics of the novel developed methods.

Penicillins are another class of antibiotics widely used for animals. In 2015, Lirio et al. developed an aluminum-based MOF-polymer (MIL-53) monolithic column for the in tube solid phase micro-extraction (SPME) of penicillins from river water and milk samples. The material was synthesized by mixing aluminum nitrate nonahydrate and 1,4-benzenedicarboxylic acid. A 0.8 mm inside diameter (I.D.) capillary tube was pretreated with NaOH, water, methanol and a mixture of 3-trimethoxysilyl-propyl methacrylate/methanol, washed and dried prior to the filling. Then, the Al-MOF material was suspended in a mixture of methacrylate-based monomers and azo-bis-isobutyronitrile prior to vortex mixing, sonication and degassing and the column was filled. Subsequently, the column was suspended in water and the filling was polymerized in situ with microwave assistance. Milk samples were treated with acetonitrile prior to their loading to the column and for the solid phase extraction procedure the optimized parameters were: sample matrix at pH 3, 200 μL desorption volume using methanol, 37.5% of MOF in a 4-cm column length, flow rate of 0.100 mL min^−1^, column conditioning with 0.5 mL methanol (MeOH) and 0.5 mL pH 3 phosphate buffer saline solution, sample volume 2 mL, column washing with 0.5 mL pH 2 phosphate buffer saline. With this procedure high extraction efficiency was succeed not only due to the π-π interactions between the absorbent and the analytes but also due to the breathing ability of MIL-53. In addition, three other aluminum-based MOFs; DUT-5, CYCU-4, MIL-68, were prepared according to the optimized condition for MIL-53-polymer in order to find out which one is more suitable for the extraction procedure. The method efficiency was satisfactory, with recoveries ranging from 80.8% to 90.9% and a limit of detection between 0.06–0.26 μg/L [32].

A novel on-line solid-phase extraction application of MOF material ZIF-8, which is a zeolite imidazole framework was proposed by Yang et al., for the determination of tetracyclines in milk samples by HPLC. For this purpose, 390 mg of the material were packed into a stainless-steel column (3 cm × 4.6 mm I.D.) which was coupled on the HPLC injector valve, in order to replace the sample loop. The extraction was achieved at a flow rate of 3 mL min^−1^ for 10 min with the use of a flow-injection system. The milk was treated with McIlvane/ethylenediaminetetraacetic acid (EDTA) buffer and the mixture was centrifuged. For the preconcentration “load” valve position was used, while unwanted sample water was going to waste. Then, with the use of “inject” position the HPLC mobile phase (10% MeOH-20% acetonitrile (ACN)-70% of 0.02 M oxalic acid solution) was pumped in the backflush mode to elute the analytes from the SPE column into the analytical column for the HPLC analysis. The proposed method was the first online SPE method that used MOF material as absorbent for milk samples. Εnhancement factors of 35–61 were obtained, recoveries were between 70.3% and 107.4% and limits of detection ranged from 1.5–8.0 μg/L. Good validation results were obtained which indicates that the developed method can be used for preconcentration of multiple analytes from complex samples [33].

Lan et al. in 2016 published an interesting approach for the determination of estrogens in milk by solid phase micro extraction including a novel fiber coating synthesis. For this purpose, cathodic electrodeposition (CED) was used for the in situ synthesis of a MOF-5 coating material for an SPME fiber (average thickness 12.5 μm). The fiber was used for the extraction of estrogens from milk samples that had been previously treated with acetonitrile for ultrasound-assisted extraction, dried and been reconstituted in n-hexane. The fiber was immersed in 5 mL of the extract for 30 min under mixing at 1000 rpm for the extraction and then it was rinsed with hexane for 10 s and immersed in an SPME-HPLC coupling device for 10 min to desorb the analytes in 60 mL of methanol prior to the injection to the HPLC system. After the whole procedure the fiber was conditioned with methanol for 10 min. For the estrogens low LODs (0.17–0.56 ng/mL) and recoveries ranged between 73.1% and 96.7%. Moreover, good method validation results were obtained, which shows that the fiber could be industrialized [34].

### 2.2. Beverages

A composite HKUST-1 MOF was used for the preconcentration of ultra-high-performance liquid chromatography with fluorescence detection (FLD), for the determination of polycyclic aromatic hydrocarbons (PAHs) in water and fruit tea infusions [35]. Tea beverages contain caffeine, as well as other xanthine derivatives like theobromine and theophylline. Phenolic compounds including phenols, phenolic acids, phenylpropanoids, flavonoids, flavones, flavonones, isoflavones, xanthones, aurones, quinines, and tannins can also be present as they can be found in tea leaves and extracted into the infusion [59]. Water was boiled, and tea was placed in water for 10 min prior to filtration and dispersive micro-extraction with the composite magnetic Fe_3_O_4_@HKUST-1 material. The interaction between the MOF material and the iron (II, III) oxide (Fe_3_O_4_) magnetic nanoparticles was achieved with the application of vortex mixing for 30 s. Afterwards, the material was placed together with the tea sample for the ultra-sound assisted extraction of the analytes for 5 min and a strong external magnetic field was used to separate the material. For the elution an aliquot of 0.5 mL of acetonitrile was used together with vortex mixing, and the procedure was repeated thrice. The eluent was filtered, dried under nitrogen and reconstituted in the mobile phase prior to the injection to the UHPLC system. LODs were 0.8 ng/L and recoveries for fruit tea were on average 75% [35].

A combination of two microextraction techniques was implemented by Lu et al. for the extraction and preconcentration of pyrethroids in water and two different tea samples prior to gas chromatography-electron capture detection (GC-ECD) detection. Therefore, a magnetic solid phase extraction coupled with dispersive liquid-liquid microextraction, with solidification of a floating organic drop (MSPE-DLLME-SFO) procedure was developed that included the MOF material MIL-101(Cr)-based for the MSPE step. Firstly, the tea was added in boiling water for 10 min and the extract was filtered. For the MSPE procedure, 10 mg of the MOF material was dispersed in a conical flask containing 50 mL of the sample together with ultrasonic irradiation for 10 min. A magnet was used to transfer the pyrethroid-absorbed magnetic material into a centrifuge tube and 600 μL of methanol was added during ultrasonic mixing for 2 min to desorb the analytes. Magnetic separation took place and the eluate was injected rapidly in a tube containing 5 mL of water. Methanol was used as the elution solvent for the MSPE procedure and the disperser solvent for the DLLME-SFO. Immediately, 50 μL of 1-dodecanol was injected into the solution and mixed in vortex for 10 s to form a cloudy solution with entire dispersion of 1-dodecanol droplets which extracted the analytes within seconds. The alcohol was separated from the mixture by centrifugation and solidification of the floating organic drop in an ice bath. As the last step, the 1-dodecanol became liquid again in room temperature and was injected in the gas chromatography system. The developed composite extraction procedure showed high sensitivity (LODs less than 0.015 ng/mL, LOQs less than 0.050 ng/mL), satisfactory precision and recovery (78.3–103.6%) [36].

Wang et al., synthesized a MOF and GO hybrid composite for solid-phase extraction and preconcentration of luteolin (i.e., a common flavonoid) from tablets and chrysanthemum tea samples. The Cu_3_(BTC)_2_/GO material was made using the solvothermal method by mixing copper nitrate trihydrate and benzene-1,3,5-tricarboxylic acid in *N*,*N*-dimethylformamide (DMF) and by adding the GO powder. Tea samples were transferred into a beaker together with 10 mL ethanol for 20 min with ultrasonic, filtration took place and the extract was diluted to 50 mL of ethanol. For the luteoline extraction 10 mL sample solution was transferred to a beaker and the pH of the solution was adjusted to 6. Then, 15 mg of sorbent was added, and the solution was stirred for 20 min for the adsorption. After that the suspension was separated and the sorbent was shaken with 2.5 mL ethanol and phosphate buffer solution mixture to elute the analytes. The eluent was placed into an electrochemical cell for subsequent detection by square wave anodic stripping voltammetry. Limit of detections were of 7.9 × 10^−10^ mol/L and recovery values for chrysanthemum tea were 99.4–101.0%. Moreover, good adsorption capacity was obtained by the novel material for the extraction procedure and the method was efficient for the enrichment of the sample [37].

In 2016, Wu et al. published a method for the determination of Hg(II) in tea and mushroom samples based on MOF as solid phase extraction sorbents. The MOF material which was used in this study was JUC-62 and it was made of 3,3′5,5′-azobenzenetetracarboxylic acid and copper nitrate trihydrate. Tea samples were dried and digested with nitric acid prior to dilution to 25 mL with deionized water and pH value was adjusted to 6–7. Both static and kinetic adsorption conditions were studied. For the static adsorption experiment 5 mg of the material were added to 5 mL of sample solution. The suspension was then shaken at room temperature for one hour, and then centrifuged. Accordingly, for the dynamic adsorption study, shaking was separately applied for a period of time. After adsorption, the suspension was centrifuged and the crystals were dispersed in 5 mL of acetate buffer together by shaking at room temperature for 10 min. Then, the suspension was centrifuged and the concentration of desorbed mercury was measured by atomic fluorescence spectrometry (AFS). Recovery values for tea samples were in average 93.3%. The static adsorption isotherm exhibited excellent adsorption capacity. The obtained results indicate that the material is promising for the sample preparation for the determination of Hg^2+^ [38].

### 2.3. Fish

Lipids and proteins constitute the main components of fish tissues. The exact chemical composition of fish depends on the fish species as well as on age, season, sex and environment [60]. For fish sample preparation, many MOF materials are reported in the literature. Lin et al. developed a Fe_3_O_4_-MOF-5(Fe) composite magnetic material, which was used as a coating for a Nd-Fe-B permanent magnet for stir bar sorptive extraction (SBSE) of six polychlorinated biphenyls, a class of toxic persistent organic pollutants. SBSE is a sensitive equilibrium technique with good reproducibility, which is generally classified as a “green analytical technique”, because it is considered to be solvent-free, or uses very low volumes of organic solvents. Due to the high coating amount, SBSE shows good recovery and extraction capacity and it has been used for the analysis of different complex matrices and a wide class of analytes. Lin et al. synthesized four different MOF materials (MIL-101(Cr), MOF-5(Zn), ZIF-8, and MOF-5(Fe)) and used them as coating of SBSE bar and found that Fe_3_O_4_-MOF-5(Fe) material had the best extraction efficiency. The material was synthesized by mixing amine-functionalized Fe_3_O_4_ nanoparticles with terephthalic acid and ferric nitrate nonahydrate and 40 mg of it were used as the coating for the SBSE procedure. For the fish sample preparation, the samples were homogenized, extracted with n-hexane under sonication, followed by filtration. Then the filtrate was loaded onto the cartridge, it was dried and finally dispersed in deionized water. Afterwards, 20 mL aqueous sample was placed into a vial into which the stir bar was immersed, and extraction took place at 700 rpm for 30 min. Desorption of the analytes was succeed with 2.5 mL of n-hexane in an ultrasonic bath within 3 min. The elution solution was dried under nitrogen atmosphere and was further diluted into 1 mL of isooctane for the Gas Chromatography-Mass Spectrometry (GC-MS) analysis. During extraction process optimization, it was found that the best efficiency is achieved at pH 7 and with a sodium chloride (NaCl) concentration of 10% *m*/*v*. The developed method was simple and sensitive and showed good linearity, low detection limits (0.061–0.096 ng/g) for the six studied polychlorinated biphenyls and recovery values more than 80% [39].

The same working group published in 2016 another analytical process for the selective enrichment and determination of polychlorinated biphenyls in fish samples using aptamer-functionalized stir bar sorptive extraction prior to GC-MS analysis. Therefore, immobilization of aptamer, which could recognize two analytes took place on a MOF-5 material that was fabricated by electro-deposition. For the extraction, the bar was placed into sample solution for 1.0 h. Desorption was performed in 5 mL of dichloromethane/glycine-hydrochloric acid (HCl) buffer (*v*/*v*, 1/10) under stirring. Limit of detections ranged from 0.003 to 0.004 ng/mL and recoveries were higher than 80%. Because of the high surface area and high selective recognition of aptamer towards the biphenyls, the prepared MOF based coating showed high selectivity and it can be used for other target analytes by changing the aptamer [40].

In 2013, Hu et al. used a Fe_3_O_4_-MOF-5(Zn) composite material for the determination of polycyclic aromatic hydrocarbons and gibberellic acids (GAs) in environmental, plant and food samples prior to GC-MS and liquid chromatography-tandem mass spectrometry (LC-MS/MS) analysis. For the synthesis of the MOF-5 material terephthalic acid and zinc diacetate hydrate was used together with amine-functionalized Fe_3_O_4_ nanoparticles. For the preparation of fish samples, the fish were ground and then extracted with Florisil and a mixture of *n*-hexane and dichloromethane (1:1, *v*/*v*) was added. For the MSPE procedure for the determination of the gibberellic acids, a quantity of 30 mg of the composite material was added to 10 mL of extracted fish sample dissolved in n-hexane, which was placed into ultrasonic bath for 30 s and then shaken on a rotator. The MOF material was collected by applying a magnet to the outer wall of the vial and elution of the analytes took place with 1.0 mL of acetonitrile containing 1% formic acid under ultrasound. Afterwards, the supernatant was evaporated under N_2_ atmosphere and re-dissolved in 100 μL of formic acid in water (0.1%, *v*/*v*) prior to LC-MS/MS analysis. Accordingly, for the enrichment of the polycyclic aromatic hydrocarbons, a portion of 50 mg of the MOF material was added to 25 mL of the extracted solution, the same procedure was followed, thus desorption was performed with 0.25 mL of acetone prior to GC-MS analysis. This method was proved to be ideal both for polar and for non-polar analytes and it showed good sensitivity, linearity, repeatability, low detection limits (0.91–1.96 ng/L for PAHs and 0.006–0.08 μg/L for GAs) and satisfactory recoveries (66.4–120.0% for PAHs and 90.5–127.4% for GAs) [41].

In 2018, Zhou et al. used a magnetic mesoporous metal-organic framework-5 for the effective enrichment of malachite green and crystal violet; two triphenylmethane dyes in fish samples. For the synthesis of the material polyethyleneimine functionalized Fe_3_O_4_ nanoparticles were mixed with zinc acetate dihydrate and terephthalic acid. Fish samples were treated with acetonitrile together and the mixture was sonicated for 10 min followed by centrifugation. The extract was dried and dissolved in 1 mL of ethanol and 10 mg of the magnetic MOF composite material was added for the MSPE procedure combined with shaking for 40 min. A magnet was used for separation of the material and analytes were extracted in methanol containing 1% formic acid, and the desorption time was 20 min prior to UHPLC-MS/MS analysis. Detection limits were 0.30 ng/mL for malachite green and 0.08 ng/mL for crystal violet, while recoveries ranged from 83.15% to 96.53%. The developed MOF material can be further studied for the adsorption of these compounds from various complex matrices [42].

HKUST-1 (MOF-199) material have been extensively studied for the determination of heavy metals from fish samples prior detection with atomic absorption spectroscopy (AAS). In 2012, Sohrabi et al. published an analytical method for the determination of Cd(II) and Pb(II) with flame atomic absorption spectroscopy (FAAS) using this magnetic MOF. For this purpose, Fe_3_O_4_-pyridine conjugate was prepared and mixed with copper(II) nitrate trihydrate and trimesic acid. For the sample preparation the fish samples were digested with nitric acid. For the MSPE procedure, 30 mg of the magnetic sorbent was added into the solutions (pH 6.3) and the mixture was stirred for 14 min to extract heavy metal ions completely. As final step 6.0 mL of 0.01 mol L^−1^ NaOH in EDTA solution was used for the elution. The elution step required 16.5 min. For Cd(II) limit of detection were 0.2 μg/L and recoveries in real samples ranged between 95–117%, while for Pb(II) 1.1 μg/L and recoveries in real samples ranged between 92.8–103.3% [43].

In 2013, Taghizadeh et al. developed a method for the determination of Cd(II), Zn(II), Ni(II), and Pb(II) ions with FAAS using the same MOF material as absorbent. For the preparation of the material, Fe_3_O_4_ nanoparticles were modified with dithizone and a copper-(benzene-1,3,5-tricarboxylate) MOF made after the reaction of trimesic acid and copper(II) nitrate trihydrate. For the sample preparation fish samples were digested with nitric acid. For the MSPE procedure, 25 mg of the magnetic sorbent was added into the solutions (pH 6.4) and the mixture was stirred for 13 min to extract the metal ions completely. As final step 7.8 mL of 0.9 mol L^−1^ thiourea in 0.01 mol L^−1^ NaOH solution were used for the elution that required 19 min. Limits of detection were found to be 0.12 ng/mL for Cd(II), 0.39 ng/mL for Zn(II), 0.98 ng/mL for Ni(II), and 1.2 ng/mL Pb(II) and recovery values were more than 90%. Potentially interfering ions does not affect the determination of the Cd(II), Zn(II), Ni(II) and Pb(II) ions [44].

In 2016 Tadjarodi and Abbaszadeh developed a method for the determination of Hg(II) with cold vapor AAS. For this purpose, Fe_3_O_4_ nanoparticles were modified with 4-(5)-imidazole-dithiocarboxylic acid and then reacted with trimesic acid and Cu(II) acetate to form the metal-organic framework HKUST-1 (MOF-199). The material was used as the adsorbent for the determination Hg(II) with MSPE. Therefore, after digestion with HNO_3_ a portion of 24 mg of the MOF material was added to the aqueous sample the pH of which was adjusted to 6.0 and the mixture was stirred for 8 min. An external magnetic field was applied in order to collect the material and elution took place with the use of 3.5 mL of 1.1 mol L^−1^ of thiourea solution under shaking for 11 min. With the developed method LODs was 10 ng/L and LOQs was 40 ng/L and satisfactory recovery (95–102%) was obtained [45].

In 2016, Ghorbani-Kalhor used HKUST-1 that was modified with magnetic nanoparticles carrying covalently immobilized 4-(thiazolylazo) resorcinol (Fe_3_O_4_@TAR) for the determination of Cd(II), Pb(II), and Ni(II) ions with FAAS from seafood (fish and shrimps) and agricultural samples. Fish samples were digested with nitric acid and then 50 mg of the material were added to the sample (pH 6.2, 10 min), for the MSPE procedure. Elution was achieved with 5 mL of a 0.6 mol/L EDTA solution for 15.2 min. LOD values ranged from 0.15 to 0.8 ng/mL [46].

All the four above mentioned methods, which included metal ions determination, were found to be characterized as simple, fast, reproducible, and selective method and the developed sorbent shows high sorption capacity, low limit of detection and high enrichment factor. Moreover, their breakthrough volume was 1000 mL and potentially interfering ions did not affect the determination of the examined metal ions. The LOD values were 0.15, 0.40, and 0.8 ng/mL for Cd(II), Ni(II) and Pb(II) ions, accordingly [42,43,44,45,46].

### 2.4. Meat, Chicken and Shrimps

Shrimp tissues contain proteins in high concentration. It also contains fatty acids (unsaturated) as well as minerals like calcium. The final shrimp tissue composition depends on the feed [61]. Shrimp samples were treated with composite HKUST-1 material for the determination of Cd(II), Pb(II), and Ni(II) ions by FAAS after digestion with nitric acid as mentioned above [46]. Sulfonamides have been also examined in shrimp samples, together with chicken and pork meat. Meat besides water contains protein and amino acids, minerals, fats and fatty acids, vitamins and other bioactive components, and small quantities of carbohydrates. Percentage composition varies according to animal species (beef, porcine, chicken, etc.) [62]. Sample preparation was achieved with a magnetic and mesoporous metal-organic framework and determination was performed by high-performance liquid chromatography. For this purpose, Fe_3_O_4_@JUC-48 material was prepared by mixing mercaptoacetic acid functionalized Fe_3_O_4_ nanoparticles, cadmium nitrate tetrahydrate and 1,4-biphenyldicarboxylic acid. Shrimps, pork and chicken samples were homogenized and placed in a centrifuge tube with acetonitrile under vortex mixing for 5 min, followed by ultra-sound assisted extraction for 30 min. Then, for the MSPE procedure a portion of 25 mg of the composite MOF material was added to the extracts and the mixture was shaken for 8 min. An external magnetic field was used to collect the material and 0.8 mL of methanol with 5% acetic acid was added into the tube and ultrasonic elution of the analytes took place in 10 min. Limit of detection ranged from 1.73 to 5.23 ng/g, recovery valued were between 76.1% and 102.6%. The developed method was successfully applied to real samples [47].

In 2017, Wang et al. published a dispersive micro-solid-phase extraction (d-μ-SPE) of three different kinds of traces of drugs in chicken breast using MIL-101(Cr)@GO composite material in microwave-assisted extraction coupled with HPLC–MS/MS detection. GO was dispersed in water and mixed with terephthalic acid and chromium nitrate nonahydrate. Then, acetonitrile was used with the following extraction conditions: 5 min extraction time at 50 °C with microwave power at 500 W. Then, 8 mg of MIL-101(Cr)@GO was used for the extraction of analytes in combination with vortex mixing for 10 min and centrifugation for 5 min. Elution was achieved with 1 mL of methanol and sonication in 15 min. As last step centrifugation took place for 5 min to separate the material. The liquid was evaporated and re-dissolved in the mobile phase for the HPLC analysis. The process reduced the consumption of organic solvent and was simple to operate. Good precision results were obtained, LODs were between 0.08 and 1.02 ng/kg and recoveries ranged from 88.9% to 102.3% [48].

### 2.5. Fruits and Vegetables

The main chemical components of fruit and vegetables include carbohydrates, dietary fiber, enzymes, protein, fat, minerals, vitamins, phenolic acids and carotenoids. However, the exact chemical composition of different fruit and vegetables depend greatly on the ripening stage, the cultivation conditions as well as the postharvest conditions [63].

In 2010, Barreto et al. indicated that lettuce samples can also be treated with MOF materials for sample preparation. A three dimensional ∞[(La_0.9_Eu_0.1_)_2_(DPA)_3_(H_2_O)_3_] material was used for the matrix solid phase dispersion (MSPD) of pesticides from lettuce prior to GC-MS determination. The material was prepared by mixing La_2_O_3_, Eu_2_O_3_, pyridine-2,6-dicarboxylic acid and water under pressure at 180 °C for three days. Lettuce samples were diced with a knife and placed into a glass mortar with 0.5 g of the material and the pestle was used for homogenous mixing. After that the mixture was placed in a 100 × 20 mm i.d. polypropylene column packed with glass wool together with anhydrous magnesium sulfate and activated carbon. Then a volume of 30 mL of acetonitrile was introduced into the column to elute the analytes. The eluent was collected and concentrated in a vacuum evaporator to a volume of 1 mL and 1 μL of it was directly analyzed with gas chromatography. LOD values were found to be 0.02–0.05 mg/kg, LOQ values were found to be 0.05–0.10 mg/kg, while recoveries ranged from 78% to 107%. Good validation results were obtained, and the developed method can be useful in screening protocols for the determination of pesticides by GC [49].

In 2018, Yan et al. synthesized electrospun UiO-66/polyacrylonitrile nanofibers and used them as adsorbent for pipette tip solid phase extraction of phytohormones in watermelon and mung bean sprouts. The vegetables were cut and ground to form fine powder with liquid nitrogen, followed by extraction with methanol with the use of ultrasonic radiation for 40 min. After centrifugation the obtained liquid could be treated with the MOF material. UiO-66 material was made by mixing terephthalic acid and zirconium(IV) chloride and polyacrylonitrile was added to the spinning solution to obtain the composite material. The nanofibers (5 mg) was placed in a 200 μL pipette-tip that was inserted into a 1.0 mL pipette-tip. The tip was activated with methanol and water, and 1.0 mL of the sample solution was loaded into it to adsorb the analytes. The pipette tip cartridge was washed with 15% methanol-water and a solution of 90% acetonitrile-ammonia was used for the elution. The eluent was dried under nitrogen and re-dissolved in the mobile phase for HPLC analysis. For the four phytohormones recoveries ranged from 88.3% to 105.2%. Limit of detection values were low (0.01 ng/mL to 0.02 ng/mL) and the method was found to be reliable, which indicates that it can be used for real samples analysis [50].

In 2016, Liu et al. developed a zirconium(IV)-based metal-organic framework (UIO-67) and used it as sorbent in dispersive solid phase extraction of plant growth regulator from fruits (pear, apple, grapefruit, orange and grape) prior to HPLC fluorescence detection. Zirconium tetrachloride and 4,4-biphenyldicarboxylic acid were used to make the material, while fruit samples were homogenized and centrifuged to collect the liquid. Then, 15 mg of the material was added to the sample solution and mixed with vortex for 8 min. Centrifugation was used to separate the material from the liquid. Subsequently, 0.6 mL of methanol with 5% formic acid was added for elution with sonication, followed by fluorescence labeling of the analytes with 1-(9*H*-carbazol-9-yl) propan-2-yl-methane-sulfonate. With this method good analytical performance was achieved in combination with low detection limits (0.21–0.57 ng/mL), high recoveries (89.3–102.3%) and short extraction times [51].

A one-pot synthesis of zeolitic imidazolate framework-8/poly (methyl methacrylate-ethylene-glycol dimethacrylate) monolith coating for stir bar sorptive extraction of phytohormones from fruit samples followed by high performance liquid chromatography-ultraviolet detection has been reported on the literature. The monolithic coating was made by mixing methyl methacrylate, ethylene-glycol dimethacrylate, methanol, azo-di-isobutyronitrile, zinc nitrate hexahydrate and 2-methylimidazole. The mixture was injected in a polytetrafluoroethylene (PTFE) mold with a prepared bar inserted inside under N_2_ and remained at 60 °C for 24 h, before being taken out and aged for 8 h at 60 °C. The bar was used to extract the analytes from pear and apple samples, which were previously cut and extracted with methanol/water (85/15, *v*/*v*) with vortex mixing for 1 min and sonication for 10 min. Under the optimum parameters, the stir bar was placed into a glass vial containing the sample pH value 3.0 under stirring for 50 min. After washing the bar was immersed in a small vial with 120 μL 30 mM NaOH (dissolved in methanol) for elution followed by neutralization. The stir coating was found to be sensitive and selective towards the examined analytes. Low detection limits were obtained (0.11–0.51 μg/L), as well as good recoveries (82.7–111%) [52].

In 2018, hybrid magnetic nanocomposites based on Cu-MOFs embedded with graphene oxide (GO) were used for the sample preparation of apples, plums, grapes, cucumbers and spinach for the determination of insecticides by HPLC. Firstly, 3.0 g copper(II) nitrate trihydrate was mixed with terephthalic acid in ethanol. Then, amino-functionalized Fe_3_O_4_ microspheres were prepared and mixed with the MOF material and graphene oxide. The Fe_3_O_4_@SiO_2_-GO MOF material was prepared and 10 mg of it were added into the sample solutions obtained from the fruit samples with shaking at 15 °C for 50 min. Subsequently the material was removed with a magnet and 200 μL of methanol were added for elution in 5 min with shaking prior to HPLC-UV analysis. Preconcentration was achieved and good analytical method performance was obtained. LODs ranged from 0.30 to 1.58 μg/L, LOQs ranged from 1.0 to 5.2 μg/L and recovery values were found to be 81.2–105.8% [53].

### 2.6. Other Food Samples

Another complex food matrix category; shellfish have also been treated with MOF materials prior to LC-MS/MS determination of domoic acid, the primary amnesic shellfish poisoning toxin. For this purpose, magnetic Fe_3_O_4_@SiO_2_ microspheres were synthesized with the solvothermal method and became carboxylate terminated after treatment with glutaric acid anhydride. Then Fe_3_O_4_@SiO_2_@UiO-66 core-shell microspheres were formed with the addition of terephthalic acid and zirconium(IV) chloride. The material was used for MPSE treatment of shellfish tissue, which was previously homogenized and extracted with methanol: water (1:1, *v*/*v*). For the magnetic solid phase extraction procedure 1 mg of the magnetic MOF material was added to the extraction solution and the mixture was vortexed for 6 min. Then, the material was removed with the use of a magnet and 0.5 mL of acetonitrile containing 20% acetic acid was used for the elution in combination with vortex mixing for 5 min. The elution procedure was performed three times, followed by evaporation and re-dissolving of the eluent in the mobile phase. LOD values were 1.45 pg/mL and recovery ranged between 93.1% and 107.3%. During the optimization procedure it was found that no pH adjusting, or salt addition was needed, and the developed method was fast and efficient for the extraction of polar analytes from complex matrices [54].

In 2018, Liang et al., used in situ synthesized MOF MIL-101(Cr) functionalized magnetic particles for the determination of seven triazine herbicides in rice. Firstly, Fe_3_O_4_ nanoparticles were prepared and used for the synthesis of Fe_3_O_4_@SiO_2_-NH_2_. Then the material was treated with graphene oxide and the resulting Fe_3_O_4_@SiO_2_-GO were added to the n-hexane extract obtained from the crushed rice powder together with MIL 101(Cr) and the mixture was sonicated for 25 min to obtain the Fe_3_O_4_@SiO_2_-GO/MIL-101(Cr) composite and extract analytical targets from sample. With the use of a magnet the material was removed, and the analytes were eluted with acetonitrile prior to HPLC analysis. The MSPE procedure was optimized and good recoveries (83.9–103.5%) and low limits of detection (0.010–0.080 μg/kg) were achieved for the pesticides [55].

In 2018, Shi et al. used a magnetic Fe_3_O_4_-NH_2_@MIL-101 material for the extraction of six Sudan dyes in tomato sauce. Magnetic nanoparticles were amino-functionalized and mixed with previously prepared MOL-101. Tomato sauce was treated with acetonitrile for 1 min followed by centrifugation at 4 °C for 10 min, twice. The resulting solutions were dried and re-dissolved in 1 mL of MeOH/water (1/1, *v*/*v*) in a vial, where 3 mg of the composite MOF material were added. The mixture was shaken for 2 min and then a magnet was used to separate the material for elution with 1 mL of ethyl acetate for 10 min by vortexing, twice. The two fractions of eluent were dried and reconstituted in acetonitrile prior to HPLC analysis. The method was efficient rapid and easy to apply, low LODs were obtained (0.5–2.5 μg/kg) as well as good recovery values (69.6–92.9%) [56].

MIL-101(Cr) was also used as sorbent for the dispersive solid phase extraction of herbicides in peanuts. The herbicides were ultrasonically extracted from peanut using ethyl acetate with ultrasound radiation. The resulting solution was evaporated and reconstituted into n-hexane and 7 mg of the material were added with shaking for 5 min. Separation of the material was achieved with centrifugation and elution was achieved with acetonitrile followed by evaporation and reconstitution in methanol prior to HPLC analysis. The above-mentioned developed method was efficient for the analysis of high in fat matrices. Low LOD values were obtained (0.98–1.9 μg/kg), as well as satisfactory recoveries (89.5–102.7%) [57].

Lead has been determined in cereal, beverages and water samples, using the zirconium-based highly porous metal-organic framework MOF-545 as adsorbent for vortex assisted-solid phase extraction prior to determination with FAAS. For the material preparation, zirconyl chloride octahydrate was mixed with *meso*-tetra(4-carboxyphenyl) porphyrin in dimethylformamide (DMF). Cereal and legume samples (chickpea, bean, wheat and lentil) were dried at 80 °C and digested firstly with HNO_3_ (65%, *w*/*w*) and then with hydrogen peroxide (30%, *w*/*w*) and diluted after evaporation in water. Cherry juice was digested with the same method and mineral water was used without digestion. For the extraction procedure 10 mg of MOF-545 was added to sample solution and the mixture was vortexed for 15 min. Centrifugation was used to separate the material and elution was achieved with 2 mL of 1 mol/L HCl solution by vortex mixing for 15 min and the eluent was separated by centrifuging. High adsorption capacity achieved, in combination with low detection limit 1.78 μg/L (with a preconcentration factor of 125) and recovery ranged from 91–96% [58].

## 3. MOF-Derived Carbon Materials

**Figure 2:**
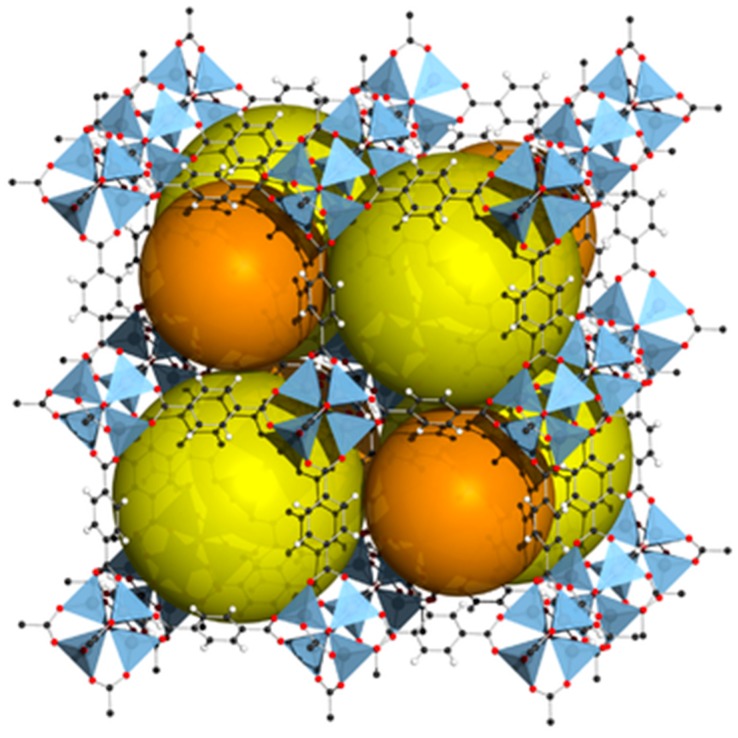
The structure of MOF-5 with orange and yellow spheres showing the pores. [Credit: Tony Boehle].

Recently, the use of magnetic nanoporous carbons derived from metal-organic framework as adsorbent for sample preparation is gaining more and more attention. Since MOFs are known for their high surface area and in combination with their mesoporous properties and the high carbon content, these materials consist a useful template to synthesize porous carbons with many potential uses, such as hydrogen storage, toxic aromatic compounds sensing, electrocatalysis, etc. [28]. As Lim et al. have found, even non-porous MOFs could result in highly nanoporous carbons [64]. In general, there are two different techniques to construct a MOF-derived nanoporous carbon. The first attempt includes impregnation of MOF carbon precursors with furfuryl alcohol as carbon source and then polymerization/carbonization as a second step. Another simpler attempt is a single-step direct carbonization [28]. MOF-5 is the most common MOF material that has been used for the synthesis of MOF derived nanoporous carbons. Figure 2 shows the structure of MOF-5 [64,65].

In 2008, Liu et al. first used A MOF as a template to make porous carbon and subsequently many applications have been reported on the literature. Those materials as well as MOFs show high surface area, large porous volume and due to their thermal stability and great electrochemical performance can be used for the same purposes as metal-organic frameworks [66,67]. Moreover, due to the presence of sp^2^ hybridized carbons they are able to form π-stacking interaction with benzene ring and aromatic compounds. As a result, those materials can be used for the adsorption of these kind of chemical compounds. When combined with magnetic precursors, MOFs can form magnetic nanoporous carbons that combine the great adsorption ability of porous carbons and handling convenience of magnetic materials [28]. Table 2 summarizes the applications of MF derived carbons for food sample preparation and some analytical details about the novel developed method.

In 2015, Liu et al., used the well-known material MOF-5 as template to form a magnetic porous material. For this purpose, MOF-5 was loaded in quartz boat and transferred in tube furnace at 80 °C for one hour in order to be carbonized under argon atmosphere at 900 °C for 6 h. Afterwards, iron(III) chloride hexahydrate and iron(II) sulfate heptahydrate was added to the porous carbon under nitrogen atmosphere at 50 °C for one hour in combination with mechanical stirring. The material was used for the determination of carbamates in apple samples with HPLC. For the sample preparation a quantity of 25.0 g of homogenized apple samples was placed in a 50 mL centrifugal tube and was centrifuged at 4000 rpm for 10 min. The supernatant was collected and filtered, and the procedure was repeated after the addition of 10 mL of water to the sediment and vortex mixing. Then, the whole extract was transferred in a conical flask for the MSPE process, where 60 mg of the MOF derived magnetic porous carbon was added and mechanical shaking was implemented.

After 25 min, the material was collected to the bottom of the flask with the use of external field (magnet) and the liquid was discarded. Elution was achieved with 200 μL of methanol and the procedure was repeated three times prior to HPLC analysis. During extraction method optimization it was found that pH value should not be higher than 6 and no salt addition is needed. The developed method showed good repeatability, linearity, precision, recovery values (89.3–109.7%) and low LODs (0.1–0.2 ng/g). Moreover, the material can be used 13 times without any loss in functionality [67].

The same research group published in 2015 an analytical method for simultaneous determination of phenylurea herbicides in grapes and bitter gourd samples, using magnetic carbon as adsorbent material for the sample preparation. The magnetic nanoporous carbon was synthesized by direct carbonization of Co-based metal-organic framework, ZIF-67. For the fabrication of ZIF-67, cobalt(II) nitrate hexahydrate and 2-methylimidazole were used. For the carbonization, ZIF-67 was heated at 150 °C for 1 h, and after that it was heated at 700 °C for 6 h under nitrogen to pyrolyze the organic species. For the sample preparation of grapes and gourd sample homogenization, centrifugation and collection of the supernatant and filtration was carried out with the same procedure as in apple sample preparation. [65,66]. For the MSPE process, a quantity of 10 mg of the magnetic porous carbon material was placed in a 100 mL flask that contained the sample solution. Shaking of the mixture took place for 25 min for the extraction and then the magnetic material was gathered to the bottom of the flask with the use of a magnet. After discarding the supernatant 0.1 mL of acetone was added to desorb the analytes for the HPLC analysis. It was found that no pH adjusting, or salt addition was required for the optimum extraction procedure. No significant loss of adsorption capacity was observed when the material was used 15 times. The developed method showed good repeatability, linearity and precision, LODs were 0.17–0.4 ng/g for the grape samples and 0.23–0.46 ng/g for the bitter gourd samples, while recoveries were 88.9–105.1% for grape sample and 89.6–104.0% for bitter gourd sample [68].

The same MOF derived magnetic nanoporous carbon was used for the determination of neonicotinoid insecticides from water and fat-melon samples by high-performance liquid chromatography ultraviolet detection (HPLC-UV). The samples were cut, homogenized and centrifuged and the MSPE procedure was similar to the above- mentioned procedure for grapes and gourd samples. Same amount of material and volume of extraction solvent was used, however shaking for the extraction took place for 20 min. Separation of the phase was carried out with the use of a magnet and after discarding the liquid phase, a volume of 0.2 mL acetone was added into the isolated MOF and vortexed for 1 min to desorb the chemical compounds. Desorption procedure was repeated one more time before HPLC analysis. During method optimization it was found that pH 6 was the ideal value for extraction and no salt addition was necessary. The MOF derived material can be used at least 15 times without functionality loss. Linearity, repeatability and method precision were good. Moreover, and LOD for the analytes in fat melon samples were 0.2–0.5 ng/g and recoveries ranged from 93.0% to 99.3% [69].

In 2016, Li et al. synthesized a Zn/Co bimetallic metal–organic framework by introducing cobalt into ZIF-8 and by direct carbonization of the resulting Zn/Co-ZIF-8 and used it as an adsorbent for the extraction of chlorophenols from water and honey tea samples prior to their determination by HPLC-UV. The MOF material was prepared by mixing cobalt(II) nitrate hexahydrate, zinc nitrate hexahydrate and 2-methylimidazole. Different molar ratios of Zn and Co complex compounds were examined and finally the ratio of Zn:Co 7:1 was chosen. Carbonization of the material took place at 900 °C for 6 h under nitrogen. For the honey tea sample preparation, the samples were diluted in a volume of 1:1 with distilled water and filtered and 15 mg of the material was added in 100 mL of the solution and the mixture was shaken for 20 min for the MSPE procedure. The material was separated from the mixture with the use of a magnet and desorption took place with 2 × 0.2 mL alkaline methanol solution and pH was neutralized with HCl solution prior to the injection to the HPLC system. As a result, a rapid, convenient, and efficient MSPE method was developed with low LOD values (0.1–0.2 ng/mL) and good recoveries (83.0–114.0%) [70].

The same year Liu et al. developed a nanoporous carbon/iron composite material MIL-53-C by one-step carbonization of the MOF material MIL-53. The novel material was used as an adsorbent for MSPE for the determination of endocrine disrupting compounds (EDCs) in fruit juices and milk by HPLC. Firstly, MIL-53 (Fe) was fabricated by mixing terephthalic acid and iron(III) chloride hexahydrate at high temperature and pressure and then carbonization was achieved by heating the material at 700 °C for 6 h under nitrogen atmosphere to pyrolyze the organic species. After juice samples were filtrated and milk samples were deproteinized and extracted with acetone, a portion of 12 mg of the material was added to the solutions for the MSPE procedure. Under optimum conditions extraction lasted for 20 min with mixing, adsorption was achieved with 0.2 mL alkaline acetone thrice and no pH adjusting, or salt addition was needed. LODs were 0.05–0.10 ng/mL for fruit juice and 0.10–0.20 ng/mL, while recovery values ranged from 92.2% to 108.3%. The method showed high adsorption capability for trace levels of EDCs and could be a promising extraction method for preconcentration of other organic compounds [71].

In 2016, Hao et al. used a metal-organic framework-derived nanoporous carbon (MOF-5-C) modified with Fe_3_O_4_ magnetic nanoparticles for the extraction of chlorophenols from mushroom samples prior to HPLC-UV determination. Excellent adsorption capacity was achieved. The carbonization of the MOF-5 nanoparticles was performed at 900 °C for 6 h under Ar. For the MSPE, 8.0 mg of Fe_3_O_4_@MOF-5-C was added to 50 mL sample solution obtained from homogenization and centrifugation of mushroom samples. The mixture was shaken on a slow-moving platform shaker for 10 min. Subsequently, the material was separated from the sample solution by putting an external magnet and 0.4 mL (0.2 mL × 2) of alkaline methanol was used for elution. The developed method was characterized as simple, fast and sensitive. Limit of detection ranged between of 0.25–0.30 ng/g, while recovery values were 85.4–97.5% [72].

In 2017, Wang et al. synthesized three-dimensional porous Cu@graphitic octahedron carbon cages that were constructed by rapid room-temperature synthesis of a Cu-based metal–organic framework (MOF) followed by further pyrolysis at 700 °C under nitrogen for the dispersive solid phase extraction of four fluoroquinolones (FQs) from chicken muscle and fish tissue prior to their determination with HPLC. The material was synthesized by the reaction of 1,3,5-benzenetricarboxylic acid and copper(II) nitrate trihydrate. Chicken and fish samples were homogenized and treated with methanol with sonication for 10 min to extract the analytes. The resulting solution was filtered, and 36.0 mg of the porous Cu@graphitic carbon cages was added into it. The mixture was vibrated for 30 min followed by centrifugation to separate the material. Elution was performed with ethanol (EtOH)/NaOH 1 mol L^−1^) (7/1, *v*/*v*) and the liquid was evaporated under nitrogen. Finally, acidic methanol was added for HPLC analysis. Low detection limits (0.18–0.58 ng/g) were obtained in combination with satisfying recoveries (81.3–104.3%). Good method performance was obtained showing great potential to further increase the applications of this novel material [73].

## 4. Conclusions

MOFs are novel composite organic-inorganic materials that have been successfully used for sample preparation of different food samples. Most applications include modification of the MOF material with magnetic nanoparticles such as Fe_3_O_4_ for the magnetic solid phase extraction of different analytes prior to their determination. In other applications the material is introduced into a column either online or offline for the extraction and pre-concentration of organic compounds in food sample matrices. However, more research has to be carried out and many factors have to be investigated for the possible automation of MOF use in sample preparation. Moreover, sensitivity of sample preparation techniques can be improved and limits of quantification (LOQs) can be obtained with the use of more sensitive analytical technique. These techniques could be inductively coupled plasma-atomic emission spectroscopy (ICP-AES) and inductively coupled plasma-mass spectroscopy (ICP-MS) for metal ions or GC-MS and LC-MS for organic compounds.

Carbonization of MOFs for the formation of MOF derived porous carbons has been also used for the sample preparation of different matrices because of the promising properties of those materials. Since there is a great variety of metal ions or clusters and organic linkers suitable to build MOF materials, many materials can be synthesized. The use of these materials or the sample preparation of food samples tend to be very promising in order to simplify the analytical procedure, to reduce the analysis cost and the organic solvent consumption.

## Figures and Tables

**Table 1:** Applications of MOF use for food sample preparation.

Matrix	Analytes	Analytical Technique	MOF Material	Sample Preparation Technique	Recovery	LODs	Reference
Milk	Sulfonamides	UHPLC-MS/MS	MIL-101(Cr)@GO	d-μSPE	79.83–103.8%	0.012–0.145 μg/L	[31]
Milk	Penicillins	UHPLC-TUV	MIL-53	In tube SPME	80.8–90.9%	0.06–0.26 μg/L	[32]
Milk	Tetracyclines	HPLC-PDA	ZIF-8	on-line SPE	70.3–107.4%	1.5–8.0 μg/L	[33]
Milk	Estrogens	HPLC-UV	MOF-5	SPME	73.1–96.7%	0.17–0.56 ng/mL	[34]
Fruit tea	Polycyclic aromatic hydrocarbons	UHPLC-FLD	Fe_3_O_4_@HKUST-1	D-μSPE	On average 75%	0.8 ng/L	[35]
Tea samples	Pyrethroids	GC-ECD	MIL-101(Cr)	MSPE-DLLME-SFO	>0.015 ng/mL	78.3–103.6%	[36]
Chrysanthemum tea	Luteolin	Square wave anodic stripping voltammetry	Cu_3_(BTC)_2_/GO	SPE	7.9 × 10^−10^ mol/L	99.4–101.0%	[37]
In tea and mushroom	Hg(II)	AFS	JUC-62	SPE	>0.58 mg/kg	On average 93.3%	[38]
Fish	Polychlorinated biphenyls	GC-MS	Fe_3_O_4_-MOF-5(Fe)	SBSE	0.061–0.096 ng/g	>80%	[39]
Fish	Polychlorinated biphenyls	GC-MS	MOF-5	SBSE	0.003–0.004 ng/mL	>80%	[40]
Fish	Aromatic hydrocarbons and gibberellic acids	GC-MS LC-MS/MS	MOF-5	MSPE	0.91–1.96 ng/L for PAHs and 0.006–0.08 μg/L for GAs	66.4–120.0% for PAHs and 90.5–127.4% for GAs	[41]
Fish	Triphenylmethane dyes	HPLC-MS/MS	MOF-5	MSPE	0.30–0.80 ng/mL	83.15–96.53	[42]
Fish	Cd(II) and Pb(II)	FAAS	MOF-199	MSPE	0.2–1.1 μg/L	92.8–117%	[43]
Fish	Cd(II), Zn(II), Ni(II), and Pb(II)	FAAS	MOF-199	MSPE	0.12–1.2 ng/mL	>90%	[44]
Fish	Hg(II)	Cold Vapor AAS	MOF-199	MSPE	10 ng/L	95–102%	[45]
Fish and shrimps	Cd(II), Pb(II), and Ni(II)	FAAS	Fe_3_O_4_@TAR	MSPE	0.15–0.8 ng/mL	NA	[46]
Shrimp samples, chicken and pork meat	Sulfonamides	HPLC-DAD	Fe_3_O_4_@JUC-48	MSPE	1.73–5.23 ng/g,	76.1–102.6%	[47]
Chicken breast	Drug traces	LC-MS/MS	MIL-101(Cr)@GO	d-μSPE	0.08 and 1.02 ng/kg	88.9–102.3%	[48]
Lettuce	Pesticides	GC-MS	∞[(La_0.9_Eu_0.1_)_2_(DPA)_3_(H_2_O)_3_]	MSPD	0.02–0.05 mg/kg	78–107%	[49]
Fruits and vegetables	Phytohormones	HPLC-FLD	UiO-66	Pipette Tip SPE	0.01–0.02ng/mL	88.3–105.2%	[50]
Fruits	Plant growth regulator	HPLC-FLD	UIO-67	d-SPE	89.3–102.3%	0.21–0.57 ng/mL	[51]
Fruits	Phytohormones	HPLC-UV	Zeolitic imidazolate framework-8	SBSE	82.7–111%	0.11–0.51μg/L	[52]
Fruits and vegetables	of insecticides	HPLC-UV	Fe_3_O_4_@SiO_2_-GO MOF	MSPE	81.2–105.8%	0.30–1.58 μg/L	[53]
Shellfish	Shellfish poisoning toxin	LC-MS/MS	Fe_3_O_4_@SiO_2_@UiO-66	MSPE	93.1% and 107.3%	1.45 pg/mL	[54]
Rice	Herbicides	HPLC-UV	MIL-101(Cr)	MSPE	83.9–103.5%	0.010–0.080 μg/kg	[55]
Tomato sauce	Sudan dyes	HPLC-DAD	Fe_3_O_4_-NH_2_@MIL-101	MSPE	69.6–92.9%	0.5–2.5 μg/kg	[56]
Peanuts	Herbicides	HPLC-DAD	MIL-101(Cr)	d-SPE	89.5–102.7%	0.98–1.9 μg/kg	[57]
In cereal, beverages and water samples	Lead	FAAS	MOF-545	Vortex Assisted SPE	91–96%	1.78 μg/L	[58]

**Table 2:** Applications of MOF-derived carbons for food sample preparation.

Matrix	Analytes	Analytical Technique	Precursor MOF Material	Sample Preparation Technique	Recovery	LODs	Reference
Apples	Carbamates	HPLC-UV	MOF-5	MSPE	89.3–109.7%	0.1–0.2 ng/g	[67]
Grapes and bitter gourd	Herbicides	HPLC-UV	ZIF-67	MSPE	88.9–105.1% for grapes, 89.6–105.0% for bitter gourd	0.17–0.4 ng/g for grapes, 0.23–0.46 ng/g for bitter gourd	[68]
Fatmelon	Neonicotinoid insecticides	HPLC-UV	ZIF-67	MSPE	93.0–99.3%	0.2–0.5 ng/g	[69]
Honey tea	Chlorophenols	HPLC-UV	ZIF-8	MSPE	83.0–114.0%	0.1–0.2 ng/mL	[70]
Fruit juice and milk	Endocrine disrupting compounds	UHPLC-FLD	MIL-53	MSPE	92.2–108.3%	0.05–0.10 ng/mL	[71]
Mushrooms	Chlorophenols	HPLC-UV	MOF-5	MSPE	0.25–0.30 ng/g	85.4–97.5%	[72]
Chicken	Fluoroquinolones	HPLC-UV	Cu based MOF	DSPE	0.18–0.58 ng/g	81.3–104.3%	[73]
